# Exploring the impact of cooking techniques and storage conditions on resistant starch levels in mung beans and its effect upon blood glucose level and lipid profile *in vivo*

**DOI:** 10.3389/fnut.2024.1424112

**Published:** 2024-09-25

**Authors:** Saloni Chauhan, Harpreet Kaur, Renuka Aggarwal, Prabhjot Kaur, Kiran Bains

**Affiliations:** Department of Food and Nutrition, Punjab Agricultural University, Ludhiana, Punjab, India

**Keywords:** cooking methods, dietary fiber, glycemic index, processed mung bean, resistant starch, storage temperature

## Abstract

**Introduction:**

Mung beans contain various antinutritional components. Processing and cooking methods can reduce these antinutritional factors and increase the availability and digestibility of nutrients. Resistant starch is also known as dietary fiber, which helps to reduce the cholesterol and glucose level in blood. It is formed during cooking and storage of food at low temperature.

**Objectives:**

This study aimed to assess the effects of cooking and storage temperature on the formation of resistant starch in processed mung bean, as well as its effect on blood glucose levels and lipid profile in humans and rats.

**Methods:**

The common cooking methods namely boiling, steaming after germination, roasting, and pressure cooking were chosen. The cooked samples were stored at different temperatures including freshly prepared within 1 h (T1), stored for 24 h at room temperature (20–22°C) (T2), kept at 4°C for 24 h (T3), and reheated after storing at 4°C for 24 h (T4).

**Results:**

The study revealed that germinated-steamed mung beans had significantly higher levels of resistant starch (27.63 ± 0.76), and lower level of glycemic index (26.28 ± 3.08) and amylose (40.91 ± 0.06) when stored at 4°C for 24 h (T3) followed by (T2), (T4), and (T1) as compared to other cooking methods (boiling, pressure cooking, and roasting). The germinated-steamed mung beans (T1) resulted in 96% decline in blood glucose parameters of rats (36 Wistar albino rats aged 2 to 3 months were selected) than the control group as observed in 28 days diet intervention (100 mg/kg resistant starch orally).

**Conclusion:**

There is a need to make people aware about the selection of appropriate cooking (steamed after germination) and storage methods (T3) to increase the RS content and to lower the glycemic index of food at domestic level.

## 1 Introduction

Worldwide, Legumes are recognized as the second most significant human food crop, after cereals. Mung bean (*Vigna radiata* L.) is a significant edible legume in several Asian nations, such as India, and is an excellent source of proteins (20–24%), carbohydrates (50–60%), crude fiber (3.8–6.2%), and lipids (0.7–1.9%) and substantial quantities of micronutrients, Mung beans contain various antinutritional components, such as hemagglutinins, phytic acid, phenolics, trypsin inhibitors, tannins, oligosaccharides, saponins, and phytic acid, in addition to their nutritional value. Processing and cooking methods can reduce these anti-nutritional factors and increase the availability and digestibility of nutrients, according to various studies. However, in addition to their antioxidant, antimicrobial, anti-inflammatory, anti-diabetic, antihypertensive, and anti-cancerous properties, these antinutritional factors also possess potent health benefit (20, 30). Mung bean seeds are predominately composed of starch, constituting a substantial proportion of the dried matter, ranging from 37 to 58% and it has been reported that mung bean starch contains a substantial proportion of resistant starch and amylose (30–45%) (7, 12, 21).

Resistant starch (RS) is comprised of alpha-linked glucose molecules unaffected by digestive enzymes in the small intestine, passing straight to the large intestine, where the gut microbiota ferments it. Based on properties that permit it to resist digestion, resistant starch is classified into four groups. RS type 1 (RS 1) is present in whole cereals and legumes and is unapproachable to enzymes that help digestion because a defensive matrix encloses it. RS type 2 (RS 2) is found in bananas (unripe), potatoes (uncooked), and maize. It has starch with high amylose and solid starch particles that are ungelatinized. RS type 3 (RS 3) are retrograded starches formed when starchy foods are cooled after cooking. RS type 4 (RS 4) is found in processed foods and is formed through cross-linking of starch chemically by adding ether and ester groups. Elongated and unbranched starch chains combine with free fatty acids to form the final type, known as RS 5. This combination forms a helical structure that makes it difficult to digest. Additionally, RS 5 includes resistant maltodextrin, a novel dietary fiber that is non-viscous and produced by deliberately rearranging starch molecules (33, 41, 42).

Resistant starch undergoes a high degree of fermentation anaerobically by resident microbiota into hydrogen, carbon dioxide, methane, and short-chain fatty acids, i.e., acetate, propionate, and butyrate, upon entering the colon. The foremost short-chain fatty acid produced from resistant starch is butyrate, which is involved in maintaining the homeostasis of the intestine. Pulses and potatoes are the best natural sources of resistant starch. The intake of resistant starch increases satiety and whole-body insulin sensitivity, reducing storage of fat, postprandial glycaemic and insulinemic responses, plasma cholesterol, and triglyceride concentrations. Resistant starch also seems to function as a prebiotic by supporting the growth of probiotic microorganisms. Regular consumption of pulses is linked with better glycemic control and lipid metabolism indicators and lower body weight (23, 35, 39, 49). Due to urbanization and exposure to social media, consumers are becoming more aware of the relationship between diet and disease. Consequently, the food industry is making efforts to produce functional foods based on different cereals, wholegrain flour, and low-glycemic-index foods.

The resistant starch content of pulses is affected by cooking and storage time and temperature; however, cooking methods, including steaming, baking, and boiling, are especially recommended to increase the amount of resistant starch in food (32). In this way, the prebiotic content of food is also improved artificially, leading to positive outcomes on health (15, 45). The amount of resistant starch in food can be increased by changing the processing parameters, such as the number of heating and cooling cycles, pH, moisture, pressure, temperature, time, freezing, and drying (18).

In the Indian diet, different pulses and legumes are used as whole grains, in the form of *dhal* (decorticated split legumes) and legume flour, in various preparations. Considering the regular intake of mung bean among Indians and many other Asians, the present study was conducted to investigate the amount of RS formed in mung bean after cooking with different techniques. Also, in today's busy world, people cook the food and store it for their convenience, the study evaluated the effect of different storage conditions including the variations in temperature and its effect on the RS content of the dhal. The effect of RS was also studied *in vivo* to investigate the efficacy of resistant starch to improve blood glucose levels.

## 2 Materials and methods

### 2.1 Procurement and cooking

**Figure 1:**
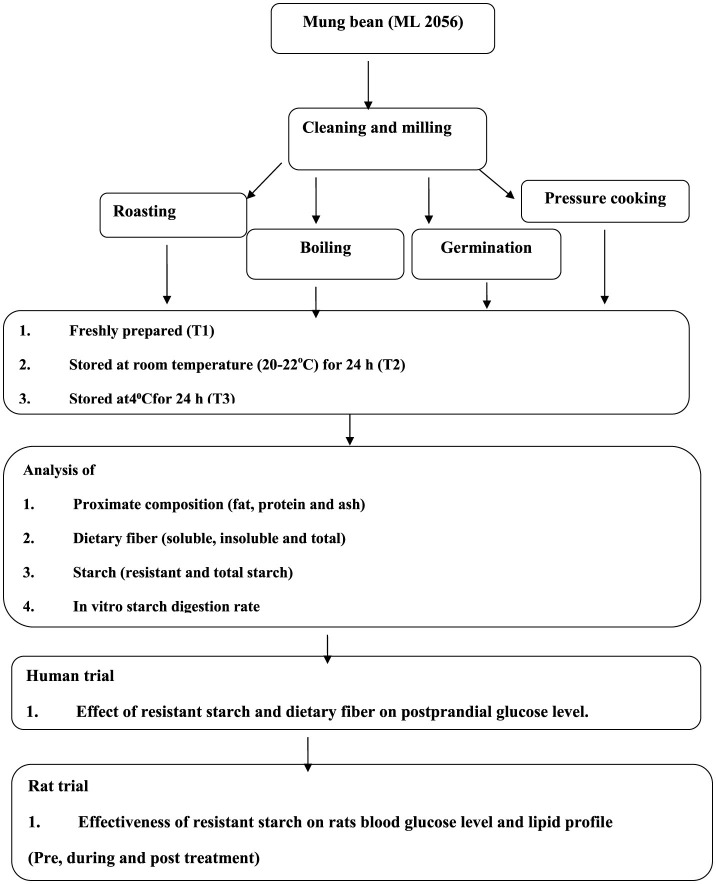
Methodology of the study.

The commonly consumed Indian mung bean variety (ML 2056) was procured from the department of Plant Breeding and Genetics, Punjab Agricultural University, Ludhiana. The grains were cleaned and ground using a sample mill with a 60-mesh size for making flour. Four common cooking methods used by North Indians, i.e., roasting for 10 min at 100°C, boiling for 30 min at 100°C, germination (48h) then steamed for 5 min, and pressure cooking (with 6 h soaking in tap water) at 100°C and at 15 lbs pressure for 8 min, were selected. These four cooking methods applied to mung bean seeds and these seeds was stored at different conditions of storage, which were considered four treatments, i.e., freshly prepared within hour (h; T1), stored for 24 h at room temperature (20–22°C; T2), stored at 4°C for 24 h (T3), and lastly reheated after storing at 4°C for 24 h (T4). After the treatments, seeds were dried and milled for making flour and used for nutritional analysis (Figure 1).

### 2.2 Nutritional analysis

The nutritional analysis of raw and cooked samples was conducted using standardized protocols to determine the levels of crude protein and crude fat (Code for crude protein AOAC 2001.11 and crude fat AOAC 945.16) (2). The Macro-Kjeldahl method was employed to calculate the nitrogen content and estimate the crude protein levels. Subsequently, nitrogen was converted into crude protein using a conversion factor of 6.25. Crude fat was estimated using soxhlet assembly. Thimbles were used to be moisture-free. The fat was extracted using petroleum ether as a solvent. The apparatus was set to a temperature of 150°C for a period of 30 min to extract fat. The beakers were placed on a heated plate to evaporate the ether, and after cooling, the beakers were weighed for fat content.

### 2.3 Dietary fiber

The total amount of dietary fiber was determined using a Megazyme total dietary fiber (K-TDFR-200A) kit. The standard method provided by AOAC (2) was also employed to examine the composition of both soluble and insoluble dietary fiber. The dietary fiber was determined using the following formula:


$$\begin{array}{l} \text{Dietary fiber}(\%)=\frac{\frac{{\text{R}}_{\text{1}}{\text{+R}}_{\text{2}}}{\text{2}}-\text{p}-\text{A}-\text{B}}{\frac{{\text{m}}_{\text{1}}+{\text{m}}_{\text{2}}}{\text{2}}}\times \text{100} \end{array}$$

Where:

R_1_ = residue weight 1 from mL, R_2_ = residue weight 2 from m_2_, m_1_ = sample weight 1, m_2_ = sample weight 2, A = ash weight from R_1_, p = protein weight from R_2_ and


$$\begin{array}{l} \text{B}=\text{blank}=\frac{{\text{BR}}_{\text{1}}+{\text{BR}}_{\text{2}}}{\text{2}}-\text{BP}-\text{BA} \end{array}$$

Where:

BR = blank residue, BP = blank protein from BR1, and BA = blank ash from BR2.

### 2.4 Total starch and resistant starch

Using a megazyme K-RSTAR test kit given by AOAC (3). A calculation of both total and resistant starches was done. In order to determine the total amount of starch, both solubilized (non-resistant) starch and resistant starch were added.

### 2.5 *In vitro* starch digestion rate

Using the methodology outlined in reference (43), *in vitro* starch digestion rate was determined. A volume of 1 mL of synthetic saliva (carbonate buffer “Sigma A-3176” Type VI-B) was used to expose 500 mg of the sample to 250 U (Unit) porcine amylase for 15.2–20 s. The solution was incubated at 37°C for 30 min after 5 mL of pepsin (breaks down proteins into smaller peptides and amino acids, it helps digest the proteins in food; 1 mL per mL of 0.02 M aq. (aqueous) HCl; obtained from gastric porcine mucosa; Sigma P-6887) was added. Prior to the pH-6 adjustment, the digest was neutralized with 5 mL of 0.02 M aq. sodium hydroxide (52.5 mL of C_2_H_3_NaO_2_ buffer at 0.2 M). The addition was made of 2 mg/mL of pancreatin (Sigma P1750 derived from porcine pancreas) and 5 mL of amyloglucosidase (It is an enzyme that can break down the α-1,4 glycosidic bonds in starch, specifically at the non-reducing ends, resulting in the production of glucose; Sigma A-7420 from *Aspergillus niger*; 28 U per mL of acetate buffer). After incubating the solution for 4 h, the glucose concentration of the digest was monitored at various intervals with an Accucheck glucometer.

### 2.6 Rapidly digestible starch and slowly digestible starch

The glucometer value taken at 15 min was used to calculate the percentage of starch digested using a specific equation (43),

Where:


$$\begin{array}{l} \text{DS}=\frac{\text{0}.\text{9}\times {\text{G}}_{\text{G}}\times \text{180}\times \text{V}}{\text{W}\times \text{S}[\text{100}-\text{W}]} \end{array}$$

GG = Reading of Glucometer (mM/L).

180 = molecular weight of glucose.

W = sample weight (g).

V = Digest volume (mL).

S = starch content (dry sample g/100 g).

M = %age of moisture (g/100 g).

0.9 = starch stoichiometric constant from glucose concentrations.

RDS% = %age of starch digested at 15 min.

SDS% = subtracting the %age of starch digested at 15 min from the %age of starch digested at 120 min.

### 2.7 Amylopectin and amylose

By using a colorimetric estimation of the amylose-iodine complex, the amylose content was determined (22). A defatted sample weighing 100 mg was combined with 1 mL of distilled ethanol in a boiling tube. Following the addition of 9 mL of 1 N sodium hydroxide to the tube, it was submerged in a water bath set to simmer for 10 min. Following the preparation of a 100-mL volume, 5 mL was transferred to a 100-ML volumetric vial. After combining this with 2 mL of iodine solution (1 g KI/500 ml distilled water) and 1 mL of 1 N acetic acid (MP Biomedicals), the mixture was left in the dark for 20 min. At 620 nm, the absorbance was measured using a blank solution that was made up of 5 mL of 0.09 N NaOH, 1 mL of acetic acid, and 2 mL of iodine solution. The volume was then set to 100 mL. The formula for amylopectin is 100 minus amylose.

### 2.8 Effect of resistant starch and soluble fiber components on postprandial glucose response by glycemic index measurement

The human supplementation was done to study the impact of processed mung beans with different treatments on the glycemic index of females. The human study was conducted in accordance with the Code of Ethics of the World Medical Association (Declaration of Helsinki). The research was carried out in adherence to the guidelines and permission provided by the Institutional Ethics Committee of Punjab Agricultural University, Ludhiana, Punjab, India. All the procedures were performed in compliance with the relevant laws. The participants were made aware of the study protocols, and informed consent was obtained prior to the study. The glycemic index of the subjects was determined using the method given by Goni et al. (19).

#### 2.8.1 Research participants and data collection

Ten female individuals aged 24–28 years were selected randomly from the girl's hostel (because of the convenience to implement study and to get unbiased results from the same participants till the end of research) at the Punjab Agricultural University in Ludhiana for measurement of blood glucose levels (Table 1). The food (containing 50 gm of carbohydrate) cooked and stored with different treatments was given in the morning after 12 h of fasting to seven different experimental groups (details below at 2.8.2), and 15 min were given to finish the meal. Blood samples were obtained using a finger-prick using a glucometer (Dr. Morphine). Blood glucose levels were assessed at specific time intervals (0, 15, 30, 45, 60, 90, and 120 min) following the consumption of 50 grams of carbohydrates from cooked Mung beans (boiled, steamed after germination, and pressure-cooked). To assess the impact of the prepared meal on blood glucose levels, 50 g of glucose was administered to a separate group of females serving as the control group. Volunteers were permitted to have 150–300 mL of water based on the food they had throughout the trial. The glycemic index was determined using a specific formula given by Wolever (49).


$$\begin{array}{l} GI\;\left(Glycemic\;index\right)=\\ \frac{Area\;under\;the\;curve\;for\;50gm\;carbohydrate\;for\;test\;sample}{Area\;under\;the\;curve\;for\;50gm\;carbohydrates\;from\;control\;\left(glucose\right)}\\ \times 100 \end{array}$$

**Table 1:** Sample size: total 10 females were given different treatment diet for 28 days.

**Group**	**Treatment diet**	**Number (*n*)**
Group-I- (G1)	Steamed after Germinated *mung bean*	10
Group-II-(G2)	Boiled *mung bean* sample after reheating	10
Group-III-(G3)	Boiled *mung bean* stored at 4°C	10
Group-IV-(G4)	Boiled *mung bean* sample after freshly prepared	10
Group-V-(G5)	Pressure cooked *mung bean* stored at 4°C	10
Group-VI-(G6)	Pressure cooked *mung bean* after reheating	10
Group-VII-(G7)	Pressure cooked *mung bean* after freshly prepared	10

#### 2.8.2 Ethical measure

The research was conducted with the approval of the Institutional Ethic Review Committee of the Punjab Agricultural University.

#### 2.8.3 Data analysis and outcome measures

Blood glucose level was assessed to calculate the glycemic index. The data was analyzed using SAS/STAT software. Mean ± Standard Deviation (S.D) for various parameters were analyzed. The data was analyzed by using analysis of Variance (ANOVA) for glycemic Index. Values were considered statistically significant at *p* < 0.01.

### 2.9 Effect of resistant starch on blood glucose level and lipid profile in *albino* male rats

The human supplementation study showed that mung beans treated with various methods had a low glycemic index. We hypothesized that foods with a lower glycemic index may positively impact diabetes treatment by potentially increasing insulin secretion or decreasing insulin sensitivity, and resistant starch as a form of dietary fiber may help to regulate lipid levels. For this, we conducted an animal trial using wistar rats to obtain authentic, real, and unbiased data. Moreover, rats are also biologically and genetically like humans, and their behavioral characteristics are strikingly similar.

#### 2.9.1 Inclusion and exclusion criteria of rats

No need of including extra rats in any group and excluding animals during the experiment and data points during analysis.

#### 2.9.2 Randomization

From the animal house and breeding center (AHBC) of Akal College of Pharmacy and Technical Education Mastuana Sahib, Sangrur (a registered breeder of CCSEA), 36 Wistar albino rats weighing between 180 and 220 g and aged 2–3 months were obtained. These rats were then randomly divided into six groups, with each group containing six rats. The research was carried out in adherence to the guidelines and permission provided by the Institutional Animal Ethics Committee (IAEC No. GADVASU/2023/1AEC/68/15). The animals were confined to enclosures, provided with water *ad libitum*, and fed commercial pellets. During the duration of the experiment, the animals exhibited a high degree of adaptability to the standard environmental conditions, which included temperature (22 ± 5°C), humidity (55 ± 5%), and 12-h light-dark cycles.

#### 2.9.3 Blinding/masking

The investigators' roles were as follows: the first investigator gave the treatment according to the randomization table. This investigator was the sole individual informed about the treatment group throughout the allocation and execution of the experiment. A second investigator was responsible for the outcome assessment whereas a third investigator (also unaware of treatment) assessed data.

#### 2.9.4 Study design

Thirty-six Wistar albino male rats aged 2–3 months with a weight of 180–220 g was divided into six groups. Group I was the control and was given AIN96M (American Institute of Nutrition Rodent Diets), i.e., the standard diet (42). Five different experimental groups, from Group II to Group VI, Mung beans processed with different treatments through oral feeding in the form of pellets were given. Foods were prepared according AIN96M (American Institute of Nutrition Rodent Diets) (Table 2). Different diets, such as normal or standard diet (STTD), whole germinated steamed mung bean diet (MSG), pressure cooked mung bean after freshly prepared diet (MPI), pressure cooked mung bean after storage at 4°C (MPRF), and pressure-cooked mung bean after reheating (MPR; detailed at 2.9.2). Rats were made diabetic by injecting them intraperitoneally with 230 mg/kg Nicotinamide (NA) in buffered saline NaCl 0.9%. After a 15-min interval, rats received a second injection of 60 mg/kg of streptozocin (STZ). Rats were given a 5% glucose solution in water for 24 h after receiving the injection to prevent hypoglycemia in groups II to VI. A window of 5 days was taken and considered as the rest period for rats. To ensure hyperglycemia, the blood glucose level, insulin level, and lipid level of the rats were measured. Blood glucose levels >200 mg/dL were considered the cutoff value for hyperglycemia. Rats were treated with a treatment diet for 28 days, and blood glucose levels were measured in the 1st, 3rd, and 4th weeks, while serum insulin and lipid profiles were measured at the beginning and end of the last week of the experiment (1, 17, 36, 37, 45, 47). Following the completion of the experiment and blood sample collection, the animals were not left untreated after the experimental procedures. The rats were euthanized by administering anesthesia (CO_2_, Isoflurane, Ketamine) method given by AVMA Guidelines for Euthanasia of animals. In accordance with university protocol, the carcasses were disposed of in a manner that ensured compliance with ethical and safety guidelines.

**Table 2:** Sample size: total 36 albino male rats with six rats in each group.

**Group**	**Treatment diet**	**Number (*n*)**
Group-I- Normal control (G1)	Standard diet	6
Group-II- Diabetic control (G2)	Standard diet	6
Group-III- Treatment group (G3)	Whole germinated steamed *mung bean* diet (MSG)	6
Group-IV- Treatment group (G4)	Pressure cooked *mung bean* after freshly prepared diet (MPI)	6
Group-V- Treatment group (G5)	Pressure cooked *mung bean* after stored at 4°C (MPRF)	6
Group-VI- Treatment group (G6)	Pressure cooked *mung bean* after reheat (MPR)	6

#### 2.9.5 Statistical methods

The data was analyzed using SAS/STAT software. Mean ± Standard Deviation (S.D) for various parameters were analyzed. The change in blood glucose was analyzed by using Tukey's test in factorial CRD while the *t*-test was used to assess lipid profile and body weight. Values were considered statistically significant at *p* < 0.01 (**Tables 6**, **7A**, **B**).

## 3 Results

### 3.1 Crude protein and fat

The crude protein content was found to be highest in germinated mung bean, where a higher value was observed in the sample having T3 (kept at 4°C for 24 h) 32.13, followed by T2 (stored for 24 h at room temperature) 31.86, T4 (reheated after stored for 24 h at room temperature) 30.46, and T1 (freshly prepared) 31.07. It was observed that all the mung bean samples cooked with boiling, roasting, and pressure cooking also had higher protein content with T3, while all the cooked mung bean samples with T1 were found to have the lowest protein content. Contrary to the protein content, all the germinated mung bean samples had a lower fat content with different treatments. The highest fat content of 1.44 g was observed in the roasted sample with T3. However, it was also seen that T3 raised the protein and fat content of the mung bean cooked with different cooking techniques (Table 3).

**Table 3:** Crude protein and fat content of mung bean (g/100 gm, dry weight basis) statistical analysis was conducted both by rows or columns and factorial CRD (completely randomized design) was applied.

**Mung bean**	**Treatment**	**T1**	**T2**	**T3**	**T4**	**LS mean**
	Boiled	24.56 ± 0.02	25.04 ± 0.02	26.04 ± 0.02	25.43 ± 0.01	25.27^C^
	Roasted	24.05 ± 0.03	24.45 ± 0.02	24.83 ± 0.08	24.12 ± 0.02	24.36^D^
	Germinated	31.07 ± 0.01	31.86 ± 0.02	32.13 ± 0.03	30.46 ± 0.02	31.38^A^
	Pressure cooked	24.87 ± 0.04	25.54 ± 0.02	26.27 ± 0.01	25.37 ± 0.01	25.51^B^
LS mean		26.13^D^	26.72^B^	27.31^A^	26.35^C^	
**Fat**						
	Boiled	1.05 ± 0.03	1.17 ± 0.01	1.27 ± 0.01	1.05 ± 0.01	1.14^C^
	Roasted	0.97 ± 0.01	1.24 ± 0.02	1.44 ± 0.02	1.12 ± 0.02	1.19^A^
	Germinated	0.65 ± 0.01	0.81 ± 0.01	0.87 ± 0.01	0.74 ± 0.03	0.77^D^
	Pressure cooked	1.07 ± 0.01	1.16 ± 0.02	1.27 ± 0.01	1.13 ± 0.01	1.16^B^
LS mean		0.94^D^	1.09^B^	1.21^A^	1.01^C^	

Effect of different cooking methods and storage temperature in the formation of protein and fat content in mung bean products. Values are mean ± SD; Mean values with different superscripts are significantly (*p* ≤ 0.05) different. T1—freshly prepared sample, T2—sample stored at room temperature for 24 h, T3—sample stored at 4°C for 24 h after cooking, and T4—reheating sample after stored at 4°C for 24 h.

### 3.2 Dietary fiber

The soluble dietary fiber content was found to be highest in boiled mung bean (6.25%), while insoluble (28.47%) and total dietary fiber (30.48%) were highest in pressure-cooked mung bean. Storing processed mung beans at various temperatures affected the dietary fiber content. The insoluble and total dietary fiber content increased with storage and was observed to be highest in products stored at 4°C (T3; 28.47, 30.48%), while the soluble fiber content was higher in freshly prepared samples (T1; 6.25%; Table 4).

**Table 4:** Dietary fiber content (soluble, insoluble, and total dietary fiber) of mung bean (g/100 gm, dry weight basis).

**Mung bean**	**Treatment**	**T1**	**T2**	**T3**	**T4**	**LS mean**
**Soluble dietary fiber**						
	Boiled	6.25 ± 0.05	6.02 ± 0.02	5.84 ± 0.02	6.22 ± 0.01	6.08^A^
	Roasted	0.15 ± 0.03	1.10 ± 0.00	0.93 ± 0.04	1.16 ± 0.02	0.84^D^
	Germinated	3.45 ± 0.04	3.20 ± 0.01	3.00 ± 0.01	3.41 ± 0.01	3.27^B^
	Pressure cooked	2.61 ± 0.01	2.21 ± 0.01	2.01 ± 0.01	3.41 ± 0.01	2.56^C^
	LS mean	3.12^B^	3.13^B^	2.95^C^	3.55^A^	
**Insoluble dietary fiber**						
	Boiled	19.85 ± 0.03	21.90 ± 0.05	23.31 ± 0.12	15.90 ± 0.22	20.24^D^
	Roasted	21.10 ± 0.06	22.85 ± 0.04	24.39 ± 0.26	20.19 ± 0.08	22.13^C^
	Germinated	22.80 ± 0.10	23.71 ± 0.12	25.26 ± 0.47	20.16 ± 0.13	22.98^B^
	Pressure cooked	26.00 ± 0.01	27.01 ± 0.01	28.47 ± 0.21	25.10 ± 0.09	26.66^A^
	LS Mean	22.44^C^	23.87^B^	25.35^A^	20.34^D^	
**Total dietary fiber**						
	Boiled	26.11 ± 0.03	27.92 ± 0.07	29.15 ± 0.14	22.11 ± 0.22	26.32^B^
	Roasted	21.25 ± 0.08	23.95 ± 0.04	25.32 ± 0.24	21.35 ± 0.07	22.97^C^
	Germinated	26.25 ± 0.14	26.91 ± 0.12	28.26 ± 0.47	23.56 ± 0.13	26.25^B^
	Pressure cooked	28.62 ± 0.02	29.22 ± 0.01	30.48 ± 0.21	28.50 ± 0.09	29.20^A^
LS mean		25.55^C^	26.99^B^	28.30^A^	23.88^D^	

Effect of different cooking methods and storage temperature in the formation of soluble, insoluble, and total dietary fiber in mung bean products. Values are mean ± SD; Mean values with different superscripts are significantly (*p* ≤ 0.05) different. T1—freshly prepared sample, T2—sample stored at room temperature for 24 h, T3—sample stored at 4°C for 24 h after cooking, and T4—reheating sample after stored at 4°C for 24 h.

### 3.3 Resistant starch

**Figure 2:**
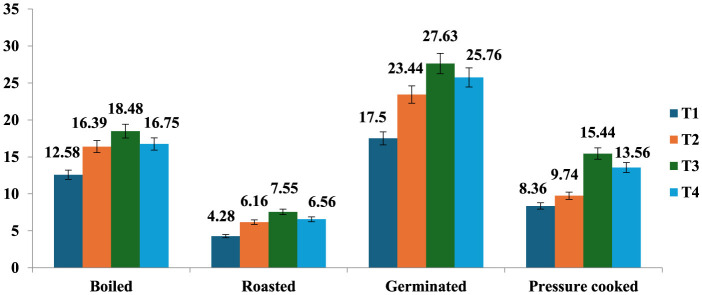
Resistant starch content (value) of processed mung bean (g/100 gm, dry weight basis). Showed different storage temperature: T1—freshly prepared sample, T2—sample stored at room temperature for 24 h, T3—sample stored at 4°C for 24 h after cooking, and T4—reheating sample after stored at 4°C for 24 h.

**Figure 3:**
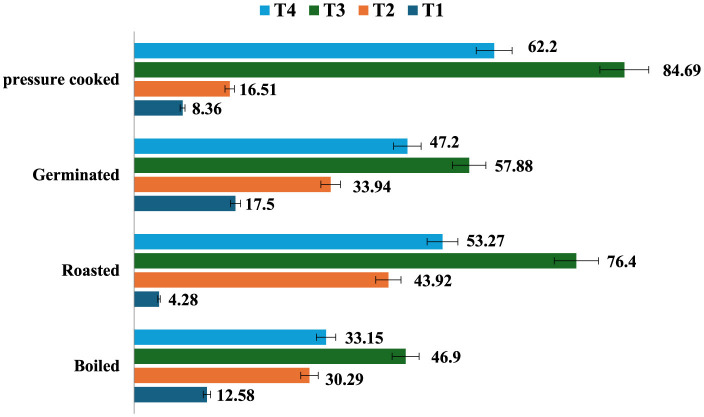
Percentage (%) change in resistant starch content among different storage condition of processed mung bean (g/100 gm, dry weight basis). Showed percentage change in resistant starch content of processed *mung bean* as compared to T1 sample as a control—T1—freshly prepared sample, T2—sample stored at room temperature for 24 h, T3—sample stored at 4°C for 24 h after cooking, and T4—reheating sample after stored at 4°C for 24 h.

The resistant starch content of raw samples (7.1%) increased after cooking except roasting, and the amount of resistant starch was found to be highest in germinated mung beans (17.5%), followed by boiling (12.58%), pressure cooking (8.36%), and roasting (4.28%). Processed mung bean stored at T3 had a higher amount of resistant starch content (27.63%), followed by T2 (23.44%), T4 (25.76%), and a lesser amount in freshly prepared products at T1 (17.05%; Figure 2). It was observed that all the treatments resulted in an increase in the RS content, while T3 resulted in the maximum percent increase in resistant starch content of all the differently cooked mung bean samples (Figure 3). The RS content increased from 4.28 to 84.69% with cooking and storage temperature and duration, indicating that time and temperature are two important factors in changing the RS content in a food sample.

### 3.4 *In vitro* starch digestion rate

The *in vitro* starch digestion rate is important in assessing a food product's ability to impact an individual's blood glucose levels. Differences in the rate and degree of starch hydrolysis affect the metabolic response to numerous starch-rich meals (13).

**Figure 4:**
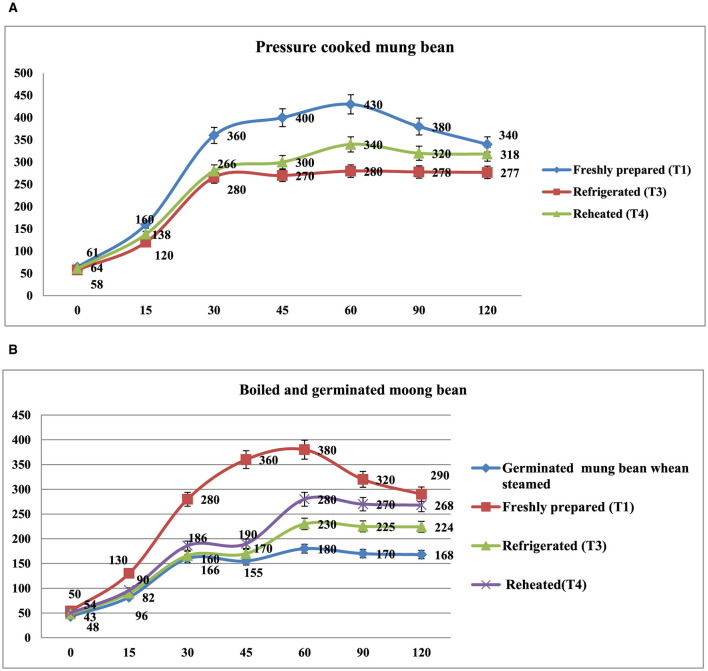
**(A)** *In vitro* starch digestion rate of pressure cooked mung bean. Showed different storage temperature: T1—freshly prepared sample, T3—sample stored at 4°C for 24 h after cooking, and T4—reheating sample after stored at 4°C for 24 h. **(B)** *In vitro* starch digestion rate of boiled and germinated mung bean. Showed different storage temperature: Germinated *mung bean* when steamed (freshly prepared), T1—immediately cooked sample, T3—sample stored at 4°C for 24 h after cooking, and T4—reheating sample after stored at 4°C for 24 h.

The *in vitro* starch digestion rate of mung bean products (boiled, steamed, germinated mung bean and pressure-cooked) affected by different storage temperatures has been shown in Figures 4A, B. The digestion rate of starch was determined at 120 min after the digestion of the food products. The germinated and boiled mung bean stored at 4°C(T3) and reheated after being stored at 4°C for 24 h (T4) had a slower digestion rate of 16.8, 27.7%, and 29.7% at 120 min compared to the freshly prepared that had completed digestion rate of 33.8% at 120 min. In pressure-cooked mung bean, the rate of starch digestion was lower in refrigerated and reheated products after being stored at 4°C for 24 h products (27.7 and 30.6%) at 120 min as compared to freshly prepared products (38.0%) at 120 min. It was observed that the starch digestion rate of pressure cooked and boiled mung bean was higher in freshly prepared products as compared to those stored at 4°C and reheated products.

### 3.5 Total starch, rapidly digestible starch (RDS %) and slowly digestible starch (SDS %)

The total starch content was observed maximum in the germinated mung bean with the highest value found in T3. On the contrary, the mean percent of rapidly digested starch and slowly digestible starch was significantly (<0.001^*^) higher in T1 pressure-cooked mung bean (22.19 and 13.94%), followed by boiled (17.10 and 6.41%), and germinated (11.65 and 1.28%). Whereas, in mung bean, the results observed that the mean percentage of slowly digested starch, when different processing methods were compared, found that the maximum value was found in pressure-cooked samples (13.94%), which was significantly (0.001^*^) higher than boiled (6.41%) and germinated samples (1.28%).

### 3.6 Amylose and amylopectin

The amount of amylose in boiled mung bean samples with T3 (38.84%) was found to be significantly (≤0.001) greater than that of T4 (37.97%), T1 (36.43%), and T2 (37.33%). Higher amylose content was also observed with T4 and cooked with different techniques i.e., roasting and pressure cooking. Contrary to this, higher amylopectin content was observed in all the samples receiving treatment T1 (Table 5).

**Table 5:** Amylose and amylopectin of mung bean (g/100 gm, dry weight basis).

**Mung bean**	**Treatment**	**T1**	**T2**	**T3**	**T4**	**LS mean**
**Amylose**						
	Boiled	36.43 ± 0.01	37.33 ± 0.18	38.84 ± 0.16	37.97 ± 0.01	37.64^B^
	Roasted	30.22 ± 0.02	31.46 ± 0.02	33.62 ± 0.02	32.04 ± 0.02	31.83^D^
	Germinated	38.83 ± 0.04	39.88 ± 0.02	40.91 ± 0.06	39.02 ± 0.01	39.66^A^
	Pressure cooked	32.81 ± 0.01	33.96 ± 0.02	35.23 ± 0.01	34.02 ± 0.01	34.01^C^
	LS mean	34.57^D^	35.66^C^	37.15^A^	35.76^B^	
**Amylopectin**						
	Boiled	63.57 ± 0.01	62.67 ± 0.18	61.16 ± 0.16	62.03 ± 0.01	62.36^C^
	Roasted	69.78 ± 0.02	68.54 ± 0.02	66.38 ± 0.02	67.96 ± 0.02	68.17^A^
	Germinated	61.17 ± 0.04	60.12 ± 0.02	59.09 ± 0.06	60.98 ± 0.01	60.34^D^
	Pressure cooked	67.19 ± 0.01	66.04 ± 0.02	64.77 ± 0.01	65.98 ± 0.01	65.99^B^
LS mean		65.43^A^	64.34^B^	62.85^D^	64.24^C^	

Values are mean ± SD; Mean values with different superscripts are significantly (*p* ≤ 0.05) different. This table showed different storage temperature: T1—freshly prepared sample, T2—sample stored at room temperature for 24 h, T3—sample stored at 4°C for 24 h after cooking, and T4—reheating sample after stored at 4°C for 24 h.

### 3.7 Effect of resistant starch and soluble fiber on blood glucose levels in human subjects

**Figure 5:**
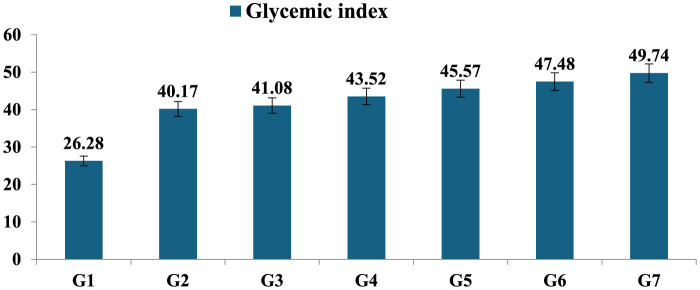
Glycemic index of processed mung bean. Showed the standard the mean glycemic index of seven *mung bean* products; 1. Steamed after Germinated mung bean (G1); 2. Boiled *mung bean* sample after reheating (G2); 3. Boiled *mung bean* stored at 4°C (G3); 4. Boiled *mung bean* sample after immediately cooked (G4); 5. Pressure cooked *mung bean* stored at 4°C (G5). 6. Pressure cooked *mung bean* after reheating (G6); 7. Pressure cooked *mung bean* after immediately cooked (G7).

The group I supplemented with germinated and steamed mung bean depicted the lowest levels of glycemic index (26.24) after the intake of the product (a sample contains 50 g of carbohydrate). The boiled and pressure-cooked mung bean samples prepared with different treatments when ingested by the experimental groups (G2–G7) showed a GI ranging from 40.17 to 49.74, with the lowest GI in the boiled T4, while in the pressure-cooked sample G5, who received food with treatment T4, the GI was 45.57. The results showed that all the samples had low GI content, which resulted in a slower rise in blood glucose levels in the participants (Figure 5).

### 3.8 Effectiveness of resistant starch of processed mung bean on blood glucose level in rats

In a 28-day trial, the effect of RS present in the treated mung bean products on the blood glucose level of rats were assessed three times, i.e., before, during, and after the completion of the diet intervention in albino male rats (Table 6). The results showed that resistant starch from various diet groups led to a decrease in blood glucose levels, although the average values did not differ significantly from either the control or diabetic control groups. In the control group (G1) and diabetic control group (G2), in all three blood assessments, no significant difference was recorded in the blood glucose level of rats. While the results of the experimental groups indicated that the maximum reduction in the blood glucose level was found in the G3 group fed on a germinated steamed mung bean diet, where their blood glucose values reduced from 278 to 144 mg/dl, The blood glucose levels of the rats belonging to G4, G5, and G6 fed on pressure-cooked mung bean with T1, pressure-cooked mung bean with T4, and pressure-cooked mung bean with T3, respectively, also reduced significantly (≤0.001) after the experiment, proving a strong effect of RS on the blood glucose levels.

**Table 6:** Effect of processed mung bean on blood glucose of rats before, during and after the experiments.

**Diet group**	**Pre-treatment**	**During-treatment (after 10 days of diet intervention)**	**Post-treatment (after 28 days of diet intervention)**	**LS mean**
G1	114.33 ± 6.86	115.00 ± 5.44	112.67 ± 4.32	114.00^F^
G2	286.50 ± 16.54	285.50 ± 13.75	283.33 ± 8.36	285.11^A^
G3	278.33 ± 11.69	175.00 ± 15.17	144.33 ± 4.63	199.22^E^
G4	294.33 ± 13.65	252.67 ± 13.95	216.33 ± 4.63	254.44^B^
G5	293.00 ± 26.25	242.00 ± 35.55	194.00 ± 4.73	243.0^C^
G6	258.50 ± 25.09	185.50 ± 8.71	169.33 ± 5.89	204.44^D^
LS Mean	256.13^A^	212.85^B^	188.28^C^	

Values are mean ± SD; Mean values with different superscripts are significantly (p ≤ 0.05) different. This table showed the standard the mean of diet group; 1. Control group (G1); 2. Diabetic control (G2); 3. Germinated mung bean fed rats (G3); 4. Pressure cooked mung bean after freshly prepared fed rats (G4); 5. Pressure cooked mung bean after reheating fed rats (G5); 6. Pressure cooked mung bean after stored at 4°C (G6) fed rats.

### 3.9 Effectiveness of resistant starch of processed mung bean on lipid profile in rats

The effect of RS on the body weight and lipid profile of the rats was also studied (Tables 7A, B). A change in body weight, plasma insulin, and lipid profile levels was observed in both control groups, i.e., G1 and G2. The change in these parameters can be attributed to the basic metabolic changes taking place in the animals due to the controlled environmental conditions. However, in all the experiment groups (G3, G4, G5, and G6), a significant (≤0.001) reduction in body weight, triglycerides, total cholesterol, and LDL levels was observed at the completion of the trial. The RS-rich diets resulted in a significant (≤0.001) increase in plasma insulin and HDL levels. The maximum increase in the plasma insulin level was found in G3, followed by G5, G6, and G4. As the plasma insulin levels increased, a similar decreasing trend in blood glucose levels was seen in G3, G6, G5, and G4. As the intervention plan was conducted under fully controlled conditions, the results clearly indicate that the decrease in blood glucose levels is purely due to the RS content of diets.

**Table 7A:** Effects of processed mung bean stored at different temperature on lipids profile of normal and diabetic rats.

**Groups**	**Body weight (grams)**			**Plasma insulin (u/ml)**			**Total cholesterol (mg/dl)**		
	**Initial (pre)**	**Final (post)**	***P*** **-value**	**Initial (pre)**	**Final (post)**	***P*** **-value**	**Initial (pre)**	**Final (post)**	***P*** **-value**
G1	175.67 ± 7.17	178.33 ± 7.31	0.02^*^	24.33 ± 0.82	24.83 ± 1.17	0.36NS	85.17 ± 3.97	85.50 ± 2.59	0.81NS
G2	171.00 ± 5.06	152.17 ± 9.02	≤0.001^*^	12.67 ± 1.63	10.00 ± 0.89	≤0.001^*^	137.33 ± 11.06	165.50 ± 5.58	0.003^*^
G3	169.67 ± 6.02	153.17 ± 4.49	≤0.001^*^	12.00 ± 1.26	31.17 ± 1.17	≤0.001^*^	134.83 ± 7.22	56.33 ± 2.34	≤0.001^*^
G4	172.50 ± 7.74	158.83 ± 7.00	≤0.001^*^	13.00 ± 1.78	17.67 ± 1.51	≤0.001^*^	138.00 ± 10.52	82.00 ± 1.41	≤0.001^*^
G5	170.33 ± 1.96	159.83 ± 5.08	≤0.001^*^	12.33 ± 1.03	23.83 ± 1.17	≤0.001^*^	134.00 ± 8.31	73.50 ± 1.87	≤0.001^*^
G6	176.83 ± 4.12	161.17 ± 5.98	≤0.001^*^	13.83 ± 1.83	23.33 ± 1.97	≤0.001^*^	134.50 ± 2.51	62.67 ± 1.75	≤0.001^*^

Values are mean ± SD; ^*^Significant at 5%; NS, non-significant. This table showed the standard the mean of diet group; 1. Control group (normal diet fed rats) (G1); 2. Diabetic control (normal diet fed rats) (G2); 3. Germinated mung bean fed rats (G3); 4. Pressure cooked mung bean after freshly prepared fed rats (G4); 5. Pressure cooked mung bean after reheating fed rats (G5); 6. Pressure cooked mung bean after stored at 4°C (G6) fed rats.

**Table 7B:** Effects of processed mung bean stored at different temperature on lipids profile of normal and diabetic rats.

**Groups**	**Triglyceride (mg/dl)**			**HDL [high density lipoprotein (mg/dl)]**			**LDL [low density lipoprotein (mg/dl)]**		
	**Initial (pre)**	**Final (post)**	***P*** **-value**	**Initial (pre)**	**Final (post)**	***P*** **-value**	**Initial (pre)**	**Final (post)**	***P*** **-value**
G1	72.67 ± 5.57	73.83 ± 6.15	0.22 NS	54.17 ± 4.58	56.17 ± 1.17	0.36 NS	23.50 ± 1.05	22.33 ± 1.03	0.20 NS
G2	126.83 ± 1.47	124.50 ± 2.66	0.084 NS	27.33 ± 1.21	27.33 ± 1.21	0.034^*^	74.33 ± 1.63	77.50 ± 3.78	0.141 NS
G3	129.67 ± 3.67	56.67 ± 2.07	≤0.001^*^	23.83 ± 2.31	67.83 ± 1.60	≤0.001^*^	70.50 ± 2.88	38.33 ± 1.86	≤0.001^*^
G4	128.17 ± 1.72	81.33 ± 1.03	≤0.001^*^	24.00 ± 1.55	53.17 ± 1.47	≤0.001^*^	68.50 ± 2.25	47.17 ± 1.47	≤0.001^*^
G5	125.83 ± 3.76	73.17 ± 1.47	≤0.001^*^	23.83 ± 1.17	56.33 ± 1.03	≤0.001^*^	68.67 ± 2.65	43.17 ± 1.17	≤0.001^*^
G6	128.67 ± 2.16	63.50 ± 1.38	≤0.001^*^	23.33 ± 1.96	62.33 ± 1.63	≤0.001^*^	66.83 ± 2.23	38.50 ± 1.52	≤0.001^*^

Each value is the mean of six observation, Values are mean ± SD. ^*^Significant at 5%; NS, non-significant. This table showed the standard the mean of diet group; 1. Control group (normal diet fed rats) (G1); 2. Diabetic control (normal diet fed rats) (G2); 3. Germinated mung bean fed rats (G3); 4. Pressure cooked mung bean after freshly prepared fed rats (G4); 5. Pressure cooked mung bean after reheating fed rats (G5); 6. Pressure cooked mung bean after stored at 4°C fed rats (G6).

## 4 Discussion

The present study was conducted with the objective of finding the optimal storage conditions and cooking method for increasing the resistant starch content of the commonly consumed mung bean in India and its effectiveness on blood glucose levels. The protein content was found to be higher in the germinated mung bean after steaming compared to other cooked samples. This could be due to the fact that germination increases the amount of protein and also improves protein quality by increasing the availability of amino acids while methods like, roasting decrease the protein content due to thermal degradation and oxidation of amino acids (29, 40). The soluble protein content leaches out during the cooking techniques in which water is used, as in the current study, pressure cooking and boiling resulted in a decrease in the protein content. Moreover, denaturation and aggregation of proteins during these processes also lead to a loss of protein content (9, 40). The different storage treatments also led to change in the protein content as during storage of food products at refrigeration temperature results in increasing or preserving the protein content due to slowing down the growth of microorganism (6). This can be clearly seen in the results found in the study as the germinated mungbean receiving T3 (kept at 4°C for 24 h) had highest protein content.

The total amount of soluble fiber is highly dependent on the cooking temperature. The high temperature breaks the linkage of glycosidic bonds in polysaccharides, which can lead to the release of oligosaccharides, increasing the percentage of soluble dietary fiber in the food. In the present study, soluble fiber was higher in freshly cooked boiled products (6.25%) than other storage treatments (4, 27, 48). The insoluble dietary fiber is >70% of TDF in the raw legumes, further increasing with different processing. Hence, processed legumes are effective in reducing glycemic responses (46). It has been reported that due to the biochemical changes occurring during cooking, the starch breaks down into soluble and insoluble dietary fiber (26). According to the present research, the pressure-cooked mungbean had a higher content of insoluble dietary fiber which could be due to the breakdown of the cell wall of the grains, which led to a higher content of insoluble fiber. Further, the pressure-cooked mungbean sample with T3 (kept at 4°C for 24 h) had the maximum insoluble fiber content, which could be due to the slowdown of the enzymatic activity and the loss of water content, resulting in an increase in the insoluble dietary fiber (31).

The highest total starch content was observed in the germinated mung bean because of biosynthesis process of starch, involving the conversion of sugars and glucose into starch molecules, mainly amylose and amylopectin (28). Resistant starch contents in different pulses and legumes that are stored at different temperature ranged from 31.60 ± 4.12 to 41.94 ± 0.43 (%w/w; mean ± SD) of the samples, this result indicates that legume starch maybe slowly digested (16). In this study, we found that resistant starch was highest in germinated mung bean stored at 4°C for 24 h. During the sprouting process, starch is rapidly hydrolyzed by the action of α- and β-amylases and α-glucosidase into dextrins and simple sugars, resulting in improved starch digestibility (15) but, the indigestible part of starch (resistant starch) remains mainly intact during germination. Moreover, during the cooling process of pulses when stored at refrigeration temperature, the de-crystalline structure of starch starts recrystallization process to become resistant to digest i.e., resistant starch. So, the rise in the RS content of the mung bean sample with T3 (kept at 4°C for 24 h) resulted in slower *in vitro* starch digestion, making it an ideal food for diabetic patients. The retrogradation process in T3 also resulted in high amylose content, as when cooked pulses and legumes are kept in the refrigerator, retrogradation occurs at a slower rate compared to room temperature, immediate cooking, and reheating. Slower retrogradation leads to the formation of a higher proportion of amylose in the starch structure (8, 48).

It was also observed that pressure-cooked mung beans with T1 had a higher starch digestion rate, which may be due to gelatinization, where the digestibility of starch is increased due to the breaking down of starch granules and making them more accessible to digestive enzymes (11, 34). In Table 8 results showed that germinated mung bean had slow starch digestion rate due to high insoluble fiber, resistant starch content, which further slows down the digestion process (5, 25). On the other hand, the total starch content in raw mung bean samples (49.23%) increased after cooking. Similarly, due to the high amount of resistant starch, insoluble fiber, and protein content, germinated mung bean when steamed had the lowest glycemic index (26.28) and glycemic load (18.14). The low glycemic index of germinated mung beans can be due to their high content of resistant starch, insoluble fiber, and protein (10). Starch is almost completely digested, but resistant starch can be digested anywhere from 5 to 7 h after a meal. The digestion process takes 5–7 h and gradually increases blood sugar levels, lowers blood sugar and insulin levels, and provides satiety for a longer period. Insoluble dietary fiber absorbs glucose molecules and prevents glucose from passing through the small intestine (25). In the human digestive system, fiber slows the increase in blood glucose and reduces glucose absorption. When it is hydrated, fiber works more efficiently to lower blood glucose levels (50).

**Table 8:** Total starch content, rapid starch digestion rate (%RDS) and slowly digestion starch rate (%SDS) in processed mung bean.

**Mung bean**	**Treatment**	**T1**	**T2**	**T3**	**T4**	**LS mean**
**Total starch**						
	Boiled	52.75 ± 0.22	55.49 ± 0.23	56.50 ± 0.13	56.20 ± 0.22	55.24^B^
	Roasted	48.44 ± 0.23	48.31 ± 0.11	47.69 ± 0.28	48.41 ± 0.19	48.21^D^
	Germinated	56.34 ± 0.14	61.06 ± 0.45	63.26 ± 0.70	62.50 ± 0.03	60.79^A^
	Pressure cooked	52.48 ± 0.12	51.79 ± 0.19	55.49 ± 0.04	56.79 ± 0.27	54.14^C^
LS mean		55.51^C^	54.16^B^	55.73^A^	55.97^A^	
**Rapid starch digestion rate (RDS %)**						
	Boiled	17.10 ± 0.09	13.24 ± 0.19	14.20 ± 0.14	≤0.001^*^	
	Pressure cooked	22.19 ± 0.08	17.94 ± 0.08	19.95 ± 0.07	≤0.001^*^	
	Germinated	11.65 ± 0.05	-	-	-	
**Slowly starch digestion rate (SDS %)**						
	Boiled	6.41 ± 0.19	2.29 ± 0.08	3.86 ± 0.02	≤0.001^*^	
	Pressure cooked	13.94 ± 0.29	9.54 ± 0.02	10.53 ± 0.20	≤0.001^*^	
	Germinated	1.28 ± 0.25	-	-	-	

Effect of different cooking methods and storage temperature in the formation of total starch, slowly starch digestion rate (SDS %), rapidly starch digestion rate (RDS %) in mung bean products. T1—freshly prepared sample, T2—sample stored at room temperature for 24 h, T3—sample stored at 4°C for 24 h after cooking, and T4—reheating sample after stored at 4°C for 24 h. ^*^Significant at 5%. Mean values with different superscripts are significantly (*p* ≤ 0.05) different.

The intervention trial results indicated a maximum reduction in blood glucose levels in the group fed on germinated mung beans. The hypoglycemic effect of germinated mung beans can be due to their higher alpha-amylase and alpha-glucosidase inhibitory activity, high resistant starch content and dietary fiber content (14, 24, 44, 49). The diet also resulted in enhanced plasma insulin and HDL (high-density lipoprotein) levels. Insulin interferes with lipolysis because lipolysis involves the synthesis of fatty acids (fats) and triglycerides (triglycerides) in adipose tissue. In diabetic rats, insulin decreased the body's ability to utilize fat for energy production through lipolysis (20) leading to an increase in the production of acetyl-CoA, which in turn increased the levels of ketones and cholesterol (17).

The values for triglyceride, total cholesterol, and LDL (low lipoprotein) were also found to be decreased (26). Resistant starch and insoluble dietary fibers also reduce lipolysis and increase the levels of Glucagon-like peptide 1 (GLP-1), peptide YY, and insulin secretion (38). GLP-1 stimulates insulin secretion and decreases glucagon secretion. Pancreatic peptide (YY) reduces appetite and increases feeling of fullness. This helps to regulate blood glucose and lipids.

## 5 Conclusion

Boiling and germination enhanced the level of resistant starch in mung beans, whereas roasting and pressure cooking reduced it. Products stored at 4°C for 24 h (T3) and at room temperature (T2) showed an increase in resistant starch content, while freshly cooked (T1) and reheated products (T4) exhibited a decrease in resistant starch. Products stored at 4°C (T3) exhibited elevated levels of insoluble dietary fiber, slowly digested starch, amylose content, and a low glycemic index and glycemic load. Research on rats showed that consuming steam-germinated mung beans is more effective in controlling the increase in blood glucose levels. Consequently, it resulted in a gradual increase in blood glucose levels, leading to extended feelings of fullness. Indians use a wide range of starchy meals. Making changes in cooking techniques and storage temperatures can result in increased content of RS in the starchy diets leading to various health advantages to the consumers.

### 5.1 Limitations

During research, weather conditions (summer/high temperature) were unsuitable for research. Because it causes the growth of microorganisms in some cooked food samples, which were stored at room temperature for 24 h and this food was unsuitable to give to the participants to consume, because it might lead to harmful health issues.

## Data Availability

The raw data supporting the conclusions of this article will be made available by the authors, without undue reservation.

## References

[1] Anderson L Dinesen B Jorgonsen PN Poulsen F Roder ME. Enzyme immune assay for intact human insulin in serum or plasma. Clin Chem. (1993) 39:578–82. 10.1093/clinchem/39.4.578 8472350

[2] AOAC. Official Methods of Analysis. 16th ed. Washington, DC: Association of Official Analytical Chemists (2000).

[3] AOAC. Official Methods of Analysis. 16th ed. Washington, DC: Association of Official Analytical Chemists (2002).

[4] Bader H Saeed F Khan MA Niaz B Rohi M Nasir MA. Modification of barley dietary fiber through thermal treatments. Food Sci Nutri. (2019) 7:1816–20. 10.1002/fsn3.1026 31139395 PMC6526641

[5] Bodinham CL Frost GS Robertson MD. Acute ingestion of resistant starch reduces food intake in healthy adults. Brit J Nutra. (2010) 103:917–22. 10.1017/S0007114509992534 19857367

[6] Boye P Zare F Pletch A. Pulse proteins: processing, characterization, functional properties and applications in food and feed. Food Res Int. (2010) 43:414–31. 10.1016/j.foodres.2009.09.003

[7] Calles T Xipsiti M Del CR. Legacy of the international year of pulses. Environ Earth Sci. (2019) 78:124. 10.1007/s12665-019-8106-6

[8] Chakraborty I Govindaraju I Kunnel S Managuli V Mazumder N. Effect of storage time and temperature on digestibility, thermal, and rheological properties of retrograded rice. Gels. (2023) 9:142. 10.3390/gels9020142 36826312 PMC9957499

[9] Dent T Campanella O Maleky F. Enzymatic hydrolysis of soy and chickpea protein with Alcalase and Flavourzyme and formation of hydrogen bond mediated insoluble aggregates. CRFS. (2023) 6:100487. 10.1016/j.crfs.2023.100487 37065430 PMC10102227

[10] Dianzhi H Qingyu Z Laraib Y Yong X Qun S. *In vitro* starch digestibility and estimated glycemic index of mung bean (*Vignaradiata L*.) as affected by endogenous proteins and lipids, and exogenous heat-processing methods. Plant Foods for Hum Nutr. (2020) 75:547–52. 10.1007/s11130-020-00845-9 32815037

[11] Ding Y Yang L Xia Y Wu Y Zhou Y Wang H. Effects of frying on starch structure and digestibility of glutinous rice cakes. J Cereal Sci. (2018) 83:196–203. 10.1016/j.jcs.2018.08.014

[12] Eashwarage LS Herath HMT Gunathilake KGT. Dietary fiber, resistant starch and *in vitro* starch digestibility of selectd eleven commonly consumed legumes (mung bean, cow pea, soyabean and horse gram) in Sri Lanka. Res J Chem Sci. (2017) 7:27–33.

[13] Edwards CH Cochetel N Setterfield L Perez-Moral N Warren FJ. A single-enzyme system for starch digestibility screening and its relevance to understanding and predicting the glycaemic index of food products. Food Funct. (2019) 10:4751–60. 10.1039/C9FO00603F 31309956

[14] Erba D Angelino D Marti A Manini F Faoro F Morreale F. Effect of sprouting on nutritional quality of pulses. Int J Food Sci Nutr. (2019) 70:30–40. 10.1080/09637486.2018.1478393 29848118

[15] Ikram A Saeed F Afzaal M Imran A Niaz B Tufail T. Nutritional and end-use perspectives of sprouted grains: a comprehensive review. Food Sci Nutr. (2021) 9:4617–28. 10.1002/fsn3.2408 34401108 PMC8358358

[16] Ezugwu EC Nwosu E Omeje KO Ubani CS. Estimation of resistant starch, non-resistant starch and total starch of unprocessed foods sourced within Nsukka Town. EAS J Nutr Food Sci. (2020) 2:159–60. 10.36349/easjnfs.2020.v02i04.001

[17] Friedwald WT Levy RI Fredrickson DS. Estimation of the concentration of low density lipoprotein cholesterol in plasma, without the use of preparative centrifuge. Clin Chem. (1972) 18:499–502. 10.1093/clinchem/18.6.499 4337382

[18] Fuentes-Zaragoza E Riquelme-Navarrete MJ Sanchez-Zapata E Perez-Alvarez JA. Resistant starch as functional ingredient: a review. Food Res Inter. (2010) 43:931–42. 10.1016/j.foodres.2010.02.004

[19] Goni I Garcia A Saura-Calixto F. A starch hydrolysis procedure to estimate glycemic index. Nutr Res. (1997) 17:427–37. 10.1016/S0271-5317(97)00010-9

[20] Grewal A Jood S. Effect of processing treatments on nutritional and antinutritional contents of green gram. J Food Chem. (2006) 30:535–46. 10.1111/j.1745-4514.2006.00080.x

[21] Hall C Hillen C Robinson JG. Composition, nutritional value, and health benefits of pulses. Cereal Chem. (2017) 94:11–31. 10.1094/CCHEM-03-16-0069-FI

[22] Juliano BO Perez CM Blakeney AB Castillo DT Kongseree N Laignelet B. International cooperative testing on the amylose content of milled rice. Starch-Starke. (1981) 33:157–62. 10.1002/star.19810330504

[23] Kendall CWC Emam A Augustin LSA Jenkins DJA. Resistant starches and health. J AOAC Inter. (2004) 87:769–74. 10.1093/jaoac/87.3.769 15287678

[24] Liyanage R Chathurang K Visvanathan R Jayathilake C. Hypolipidemic and hypoglycemic potential of raw, boiled, and sprouted Mung beans (*Vignaradiata* L. Wilczek) in rats. J Food Biochem. (2022) 42:12457. 10.1111/jfbc.12457

[25] Lopez HW Levrat-Verny MA Coudray C Besson C Krespine V Messager A. Class 2 resistant starches lower plasma and liver lipids and improve mineral retention in rats. J Nutr. (2001) 131:1283–89. 10.1093/jn/131.4.1283 11285339

[26] Lopez-Exposito I Ciruelos A. Dietary fiber composition of legumes and nuts: effect of processing. Food Res Inter. (2012) 48:777–85. 22624297

[27] Lou Yi T Wang K Zhuang Z Pan S Huang X. Comparative analysis of dietary fibre extract isolated from citrus juice by-products using water extraction, fermentation and enzymatic treatment methods. Advance J Food Sci Tech. (2014) 6:1058–66. 10.19026/ajfst.6.160

[28] Megat RMR Azrina A Norhaizan ME. Effect of germination on total dietary fiber and total sugar in selected legumes. Inter Food Res J. (2016) 23:257–61.

[29] Mubarak AE. Nutritional composition and antinutritional factors of mung bean seed (*Phaseolus aureus*) as affected by some home traditional processes. Food Chem. (2005) 89:489–95. 10.1016/j.foodchem.2004.01.007

[30] Omenna EC Olanipekun OT Kolade RO. Effect of boiling, pressure cooking and germination on the nutritional and antinutrients content of cowpea (*Vignaunguiculata*). J Food Agric Sci. (2016) 6:1–8. 10.23880/FSNT-16000104

[31] Pereira EJ Carvalho LM Dellamora GM Cardoso FS Carvalho JL Viana DS. Effects of cooking methods on the iron and zinc contents in cowpea (*Vignaunguiculata*) to combat nutritional deficiencies in Brazil. Food Nutr Res. (2013) 58:212–25. 10.3402/fnr.v58.20694 24624050 PMC3926463

[32] Perera A Meda V Tyler RT. Resistant starch, a review of analytical protocols for determining resistant starch and of factors affecting the resistant starch content of foods. Food Res Inte. (2010) 43:1959–74. 10.1016/j.foodres.2010.06.003

[33] Raigond P Ezekiel R Raigond B. Resistant starch in food: a review. J Sci Food Agric. (2015) 95:1968–78. 10.1002/jsfa.6966 25331334

[34] Ratnaningsih N Suparmo A Harmayani E Marsono Y. Physicochemical properties, *in vitro* starch digestibility, and estimated glycemic index of resistant starch from cowpea (*Vignaunguiculata*) starch by autoclaving-cooling cycles. Int J Biol Macromol. (2020) 142:191–200. 10.1016/j.ijbiomac.2019.09.092 31521656

[35] Reynolds A Mann J Cummings J Winter N Mete E TeMorenga L. Carbohydrate quality and human health: a series of systematic reviews and meta analyses. Lancet. (2019) 393:434–45. 10.1016/S0140-6736(18)31809-9 30638909

[36] Rice EW Roderick P Mac DRP. Determination of triglycerides. Standard method. Clin Chem. (1970) 6:215–22. 10.1016/B978-0-12-609106-9.50027-0

[37] Richmond W. Preparation and properties of a cholesterol oxidase from *Nocardia* sp. and its application to the enzymatic assay of total cholesterol in serum. Clin Chem. (1973) 19:1350–6. 10.1093/clinchem/19.12.1350 4757363

[38] Rimm EB. Vegetable, fruit, and cereal fiber intake and risk of coronary heart disease among men. J Am Med Assoc. (1996) 275:447. 10.1001/jama.1996.03530300031036 8627965

[39] Robillard N. Resistant Starch Friend or Foe? (2020). Available at: https://digestivehealthinstitute.org/2013/05/10/resistant-starch-friend-or-foe/ (accessed April 9, 2020).

[40] Sánchez-Velázquez OA Ribéreau S Mondor M Cuevas-Rodríguez EO Arcand Y Hernández Alvarez AJ. Impact of processing on the *in vitro* protein quality, bioactive compounds, and antioxidant potential of 10 selected pulses. Legume Sci. (2021) 88:1–18. 10.1002/leg3.88

[41] Santana ANL Meireles MAA. New starches are the trend for industry applications: a review. Food Publ Health. (2014) 4:229–41. 10.5923/j.fph.20140405.0422499009

[42] Snelson M Kellow N Coughlan MT. Modulation of the gut microbiota by resistant starch as a treatment of chronic kidney diseases: evidence of efficacy and mechanistic insights. Adv Nutr. (2019) 10:303–20. 10.1093/advances/nmy068 30668615 PMC6416045

[43] Sopade PA Gidley MJ. A rapid *in-vitro* digestibility assay based on glucometry for investigating kinetics of starch digestion. Starch-Stärke. (2009) 61:245–55. 10.1002/star.200800102

[44] Thilagavathi T Kanchana S. A study on the effect of millet and pulse based pasta on blood glucose and lipid profile in alloxan-induced diabetic rats. IJPCBS. (2017) 7:112–21.

[45] Trinder P. Determination of blood glucose using 4-amino phenazone as oxygen acceptor. J Clin Pathol. (1969) 22:246. 10.1136/jcp.22.2.246-b 5776563 PMC474047

[46] Veena A Urooj A Puttaraj S. Effect of processing on the composition of dietary fiber& starch in some legumes. Die nahrung. (1995) 39:132–8. 10.1002/food.19950390206 7783778

[47] Wahjuningsih SB Haslina H Marsona M. Hypolipidamic effect of high resistant starch sago and Red Bean flour-based Analog Rice on Diabetic rats. Mater Sociomed. (2018) 30:232–9. 10.5455/msm.2018.30.232-239 30936784 PMC6377926

[48] Wang S Li C Copeland L Niu Q Wang S. Starch retrogradation: comprehensive review. Compr Rev Food Sci Food Saf. (2015) 14:568–85. 10.1111/1541-4337.12143

[49] Wolever A Jenkins D. The use of the glycemic index in predicting the blood glucose response to mixed meals. Am J Clin Nutr. (1986) 43:167–72. 10.1093/ajcn/43.1.167 3942088

[50] Wood L Wilbourne J Kyne-Grzebalski D. Administration of insulin by injection. Pract Diabet Int. (2002) 19:S1–4. 10.1002/pdi.330

