# Corrigendum: Exploring the impact of cooking techniques and storage conditions on resistant starch levels in mung beans and its effect upon blood glucose level and lipid profile *in vivo*

**DOI:** 10.3389/fnut.2025.1605700

**Published:** 2025-06-12

**Authors:** Saloni Chauhan, Harpreet Kaur, Renuka Aggarwal, Prabhjot Kaur, Kiran Bains

**Affiliations:** Department of Food and Nutrition, Punjab Agricultural University, Ludhiana, Punjab, India

**Keywords:** cooking methods, dietary fiber, glycemic index, processed mung bean, resistant starch, storage temperature

In the published article, there was an error. A correction has been made to **Materials and Methods**, *Effect of resistant starch on blood glucose level and lipid profile in albino male rats*, paragraph five 2.94 Study design. After induction of diabetes, the rats were left untreated and was not included at the end of the paragraph. The sentence should have been written as “Rats were treated with a treatment diet for 28 days, and blood glucose levels were measured in the 1st, 3rd, and 4th weeks, while serum insulin and lipid profiles were measured at the beginning and end of the last week of the experiment (1, 17, 36, 37, 45, 47). Following the completion of the experiment and blood sample collection, the animals were not left untreated after the experimental procedures. The rats were euthanized by administering anesthesia (CO_2_, Isoflurane, Ketamine) method given by AVMA Guidelines for Euthanasia of animals. In accordance with university protocol, the carcasses were disposed of in a manner that ensured compliance with ethical and safety guidelines.”

The authors apologize for this error and state that this does not change the scientific conclusions of the article in any way. The original article has been updated.

